# Best therapeutic approach in metastatic hormone-sensitive prostate cancer based on disease volume: a systematic review and network meta-analysis

**DOI:** 10.1093/oncolo/oyaf386

**Published:** 2025-11-17

**Authors:** Fabrizio Di Costanzo, Chiara Mercinelli, Alessio Signori, Carlo Messina, Vincenza Conteduca, Giovanni Dima, Matteo Santoni, Luigi Formisano, Christoph Oing, Giuseppe Fornarini, Sara Elena Rebuzzi, Orazio Caffo, Giuseppe Luigi Banna, Ugo De Giorgi, Giuseppe Procopio, Francesco Montorsi, Alberto Briganti, Luca Galli, Massimo Di Maio, Andrea Necchi, Brigida Anna Maiorano, Pasquale Rescigno

**Affiliations:** Department of Medical Oncology, Università degli Studi di Napoli Federico II, Naples, Italy; Translational and Clinical Research Institute, Newcastle University, Newcastle Upon Tyne, United Kingdom; Department of Medical Oncology, IRCCS San Raffaele Hospital, Comprehensive Cancer Center, Milan, Italy; Vita-Salute San Raffaele University, Milan, Italy; Department of Health Sciences, University of Genoa, Genoa, Italy; IRCCS Policlinico San Martino, Genoa, Italy; Oncology Unit, A.R.N.A.S. Civico, Palermo, Italy; Unit of Medical Oncology and Biomolecular Therapy, and CREATE Center for Research and Innovation Medicine, Department of Medical and Surgical Sciences, University of Foggia, Foggia, Italy; Medical Oncology Unit 2, Santa-Chiara Hospital, Pisa, Italy; Medical Oncology Unit, Macerata Hospital, Macerata, Italy; Department of Medical Oncology, Università degli Studi di Napoli Federico II, Naples, Italy; Translational and Clinical Research Institute, Newcastle University, Newcastle Upon Tyne, United Kingdom; The Newcastle upon Tyne Hospitals NHS Foundation Trust, Newcastle Upon Tyne, United Kingdom; IRCCS Ospedale Policlinico San Martino, Genoa, Italy; Ospedale San Paolo, Medical Oncology, Savona, Italy; Department of Internal Medicine and Medical Specialties (Di.M.I.), University of Genoa, Genoa, Italy; Medical Oncology, Santa Chiara Hospital, Trento, Italy; Portsmouth Hospitals University NHS Trust, Portsmouth, United Kingdom; Faculty of Science and Health, School of Pharmacy and Biomedical Sciences, University of Portsmouth, Portsmouth, United Kingdom; University Oncology Unit, University of Salento Fazzi Hospital, Lecce, Italy; Genito-Urinary Medical Oncology, IRCCS Foundation Istituto Nazionale Tumori, Milan, Italy; Vita-Salute San Raffaele University, Milan, Italy; Department of Urology, IRCCS San Raffaele Hospital, Milan, Italy; Vita-Salute San Raffaele University, Milan, Italy; Department of Urology, IRCCS San Raffaele Hospital, Milan, Italy; Medical Oncology Unit 2, Santa-Chiara Hospital, Pisa, Italy; Department of Oncology, University of Turin, AOU Città della Salute e della Scienza di Torino, Turin, Italy; Department of Medical Oncology, IRCCS San Raffaele Hospital, Comprehensive Cancer Center, Milan, Italy; Vita-Salute San Raffaele University, Milan, Italy; Department of Medical Oncology, IRCCS San Raffaele Hospital, Comprehensive Cancer Center, Milan, Italy; Translational and Clinical Research Institute, Newcastle University, Newcastle Upon Tyne, United Kingdom; The Newcastle upon Tyne Hospitals NHS Foundation Trust, Newcastle Upon Tyne, United Kingdom

**Keywords:** mHSPC, darolutamide, enzalutamide, apalutamide, synchronous versus metachronous, low volume versus high volume

## Abstract

**Context:**

With new treatment strategies approved in metastatic hormone-sensitive prostate cancer (mHSPC), heterogeneity across trials hinders the physicians’ choice for first-line treatment.

**Objective:**

We conducted a systematic review and network meta-analysis to assess the efficacy of currently approved treatments for mHSPC stratifying patients according to their disease burden (high- vs. low-volume as per CHAARTED criteria) and onset of metastatic disease (synchronous vs. metachronous).

**Intervention:**

Eleven randomized controlled trials (RCTs) published until October 30, 2024 were included. Treatment regimens were grouped as triplets for combinations of docetaxel, androgen receptor pathway inhibitors (ARPIs) and androgen-deprivation therapy (ADT), separate doublets for docetaxel plus ADT, ARPI plus ADT, or monotherapy for ADT alone.

**Outcome Measurements and Statistical Analysis:**

Overall survival (OS) and radiographic progression-free survival (rPFS) outcomes were collected. OS as primary endpoint, and rPFS as secondary endpoint, were analyzed separately in high- and low-volume patients. Additional subgroup analyses accounted for timing of metastases categorized as high-volume/synchronous, high-volume/metachronous, low-volume/synchronous, and low-volume/metachronous disease.

**Evidence Synthesis:**

Triplet combinations prolonged significantly OS and rPFS in high-volume disease (*P*-score 0.99), and high-volume/synchronous disease (*P*-score 0.99). ARPI/ADT doublets performed best in low-volume patients (*P*-score 0.94), and low-volume/metachronous (*P*-score 0.99). In the high-volume/metachronous population, triplets, and doublets were equally effective.

**Conclusions:**

The results provide collective evidence for treatment selection based on disease volume and timing of metastasis with strongest survival benefits of triplets for high-volume/synchronous mHSPC patients and of ARPI doublets for low-volume disease.

Implications for Practice The findings of this systematic review and network meta-analysis support a more individualized treatment approach for patients with metastatic hormone-sensitive prostate cancer (mHSPC). Triplet therapy (androgen-deprivation therapy ADT] + docetaxel + androgen receptor pathway inhibitor ARPI]) should be prioritized in patients with high-volume synchronous disease, where it consistently demonstrated the greatest survival benefit. Conversely, for patients with low-volume or metachronous disease, ARPI-based doublets appear to offer optimal outcomes with a more favorable toxicity profile. These results advocate for routine stratification of mHSPC patients by disease volume and timing of metastasis onset to inform clinical decision-making and avoid both under- and over-treatment. Enhanced integration of molecular profiling in future trials will further refine patient selection for treatment tailoring.

## Introduction

Over the last decade, new evidence has dramatically changed the therapeutic landscape for patients with advanced prostate cancer. In 2015, two randomized phase III studies, CHAARTED and STAMPEDE (arm C), marked a paradigm shift as they reported a survival benefit of adding docetaxel to androgen deprivation therapy (ADT), which was longstanding standard of care in metastatic hormone-sensitive prostate cancer (mHSPC).^1^^,^^2^ Upfront doublet treatment improved both progression-free (PFS) and overall survival (OS).^3^^,^^4^

Shortly after, second-generation androgen receptor pathway inhibitors (ARPIs) were assessed in combination with ADT in the mHSPC setting. Abiraterone acetate first demonstrated a survival benefit in combination with ADT over ADT alone in the LATITUDE and STAMPEDE (arm G) trials.^5^^,^^6^ Subsequently, apalutamide (TITAN^7^), enzalutamide (ENZAMET^8^ and ARCHES^9^) and more recently darolutamide (ARANOTE^10^) achieved similar results as ADT combination partners.

As a consequence to the beneficial impact of doublet combinations, triplet combinations including ADT plus ARPI plus docetaxel were investigated with the idea to maximize anti–tumor efficacy through simultaneous treatment with different mechanisms of action.^11–13^

Recently, results from the ARASENS^14^ and PEACE-1^15^ trials reported superiority of further treatment intensification by combining ADT, docetaxel, and darolutamide or abiraterone acetate, over ADT plus docetaxel alone as first-line mHSPC treatment.^16^

Triplet combinations are associated with added toxicity and patient selection is key to preventing over- or under-treatment and unnecessary toxicity while achieving optimal treatment outcomes.^17–19^

Importantly, stratification of patients by metastasis onset (synchronous or metachronous) and disease burden (high or low) has prognostic implications with patients with synchronous high-volume disease showing the shortest survival.^20^ Based on subgroup analyses, such patients with highly aggressive disease appear to be those most likely to benefit from upfront triplet treatment. Nevertheless, there is still a lack of studies stratifying patients by disease volume/risk and/or onset of metastatic disease for the comparison of doublet versus triplet regimens.^11^

To deconvolute this conundrum, we carried out a systematic review and network meta-analysis of randomized clinical trials assessing different treatments for mHSPC patients, with the aim of synthesizing existing data and shedding light on the most appropriate and effective treatment options for mHSPC patients based on the volume of disease and onset of metastatic disease, as uniformly available clinical characteristics.

## Materials and methods

### Data retrieval strategies

We conducted a systematic review and network meta-analysis adhering to the Preferred Reporting Items for Systematic Reviews guidelines (PRISMA) and National Institute for Clinical Excellence (NICE) Decision Support Unit guidelines.^21^^,^^22^ (Figure S1). Embase, PubMed, and Web of Sciences databases were searched for randomized controlled trials (RCTs) on first-line systemic treatment of mHSPC (Table S1). Abstracts from scientific conferences, including the American Society of Clinical Oncology (ASCO), the ASCO Genitourinary Cancers Symposium (ASCO-GU), and the European Society for Medical Oncology (ESMO), were also screened to identify relevant results not yet fully published. Publications available up to October 30, 2024, were analyzed. The protocol for the systematic review was registered with PROSPERO (CRD42024620551).

### Inclusion and exclusion criteria

Three independent reviewers (F.D.C., C.M., and B.M.) screened the studies according to specific inclusion and exclusion criteria as mentioned below. In case of disagreement a decision was made through consultation of a fourth author (P.R.).

The following inclusion criteria were defined: (1) Phase II or III RCTs; (2) enrolling patients with mHSPC treated in the first-line setting with triplets (ADT + docetaxel + ARPI, either abiraterone or apalutamide or darolutamide or enzalutamide), doublets with ARPI (ADT + ARPI), doublets with chemotherapy (ADT + docetaxel), or ADT monotherapy, stratified per high- or low-volume as indicated in the RCTs (according to the CHAARTED criteria^2^); (3) reporting OS and/or radiographic PFS (rPFS) outcome data. Trials primarily investigating radiotherapy (RT) or radioligand therapies (RLT), single-arm studies, non–RCTs, pre–clinical studies, trials enrolling fewer than 10 patients, or publications not in English were excluded. However, we included the PEACE-1 trial, a pivotal phase III RCT evaluating systemic triplet therapy. Although RT was part of treatment for 1 arm recruiting patients with low-volume disease, data from systemic treatment arms only (ADT + docetaxel ± abiraterone) were considered in accordance with our pre–defined trial inclusion criteria (see Table S2 for full inclusion/exclusion flowchart).

### Data extraction and quality assessment

Three authors independently performed the data extraction, which included the first author’s name, publication year, study phase, patient numbers, patient demographics, treatments applied in the experimental and control arms, disease setting (volume and onset), and survival outcomes regarding OS and rPFS expressed or calculated as hazard ratios (HRs) and 95% confidence intervals (95% CIs).

The quality of the included studies was assessed using the Cochrane Collaboration Risk of Bias in RCTs (ROB-2) tool.^23^

The statistical analysis was performed with R Studio version 2023.03.0 + 386, with the netmeta package, using a frequentist framework, to synthesize direct and indirect evidence across treatment networks using a generalized linear model. The point estimates for HR and 95% CI for OS (pre–planned primary endpoint) and rPFS (pre–planned secondary endpoint) across first-line treatment regimens were generated to assess the comparative efficacy of the regimens. Treatments were grouped into 4 categories: (1) Triplet (featuring ADT + docetaxel + ARPI); (2) Doublet_ARPI (including ADT + ARPI, that is, abiraterone/apalutamide/darolutamide/enzalutamide); (3) Doublet_CHEMO (including ADT + docetaxel); (4) Mono (ADT monotherapy).

Two networks based on disease volume were created, as defined by each study included: (1) A high-volume patients’ network; (2) A low-volume patients’ network. Both were analyzed separately for OS (as primary endpoint) and rPFS (as secondary endpoint). OS was defined as the time from the start of treatment to the patient’s death of any cause. rPFS was defined as the time from randomization to radiographic disease progression or patients’ death, whichever occurred first. We used rPFS to homogenize the definition of PFS across the included studies, and excluded those reporting alternative definitions of PFS, such as clinical, biochemical, or combined PFS endpoints. Additional networks for high-volume synchronous/metachronous and low-volume synchronous/metachronous patients were explored as subgroups for OS, as only OS data were available for this analysis.

Results are presented in network diagrams, with knots representing each treatment strategy. Assuming a low heterogeneity between the studies, a fixed-effect model was adopted for each separate analysis.^24^^,^^25^ Node-splitting methods and global inconsistency models were implemented to verify the assumption that direct and indirect comparisons were consistent, and to ensure the network’s reliability. The global consistency of the network was evaluated using the design-by-treatment interaction model. Treatment effects were summarized using forest plots for direct comparisons relative to ADT monotherapy as reference treatment. League tables were constructed to present pairwise comparisons of treatment strategies, including direct and indirect HR estimates with 95% CIs. In the tables, each cell represented the HR and 95% CI for the treatment strategy in the row versus that in the column, with a HR <1 favoring the treatment strategy in the row. Heatmaps were generated to represent treatment comparisons, summarizing the league tables visually. Color gradients (from light to dark blue) represented the magnitude of HR values, with darker colors indicating higher HRs (for less favorable outcomes). Finally, treatment rankings were derived using *P*-scores, reflecting the treatment’s probability of being the most effective, based on the point estimates and the precision of HRs, with higher *P*-scores indicating higher treatment efficacy in the respectively assessed mHSPC setting.

For all statistical analyses, a *P*-value <.05 was regarded as statistically significant, and all tests were 2-sided.

## Results

### Search results and characteristics of the included studies

The electronic search identified 940 references from the aforementioned databases and conference abstracts. After duplicate removal, 871 manuscripts were screened overall, with 11 studies fulfilling the inclusion criteria and included in the network meta-analysis at the end of the selection process (Figure S2).

All included studies were phase III RCTs (Table 1). Three of the included trials or trial arms (GETUG-AFU15,^26^ CHAARTED,^3^ and STAMPEDE arm C^4^) tested docetaxel plus ADT doublet versus ADT in a total of 2261 enrolled patients, 6 trials (ARCHES,^9^ TITAN,^7^ STAMPEDE arm G,^6^ LATITUDE,^5^ and ARANOTE^10^) tested ARPI plus ADT doublets versus ADT + PBO or non–steroidal anti–androgens (NSAA, ENZAMET trial^8^), including a total of 7112 patients, and 2 RCTs, PEACE-1,^15^ and ARASENS^14^ tested triplet therapy versus ADT plus docetaxel in a total of 2479 patients. Further, additional triplet treatment data from 254 patients who received additional docetaxel were extrapolated from the ENZAMET trial, as in this trial up to 6 cycles of docetaxel administration prior to study inclusion and randomization were allowed (see Table 2 for details). Thus, the treatment strategies included in the resulting 14 datasets were: docetaxel plus ADT plus ARPI (triplets), ADT plus ARPI or docetaxel (doublets), and ADT alone.

**Table 1.** Characteristics of the included studies.

Trial	Time of accrual	First author	Randomization ratio	Study Phase	Experimental arm	Control arm	Other treatments allowed	Primary endpoint(s)
**GETUG-AFU 15^26^ ^,^ ^27^**	October 2004-December 2008	Gravis et al.	1:1	III	Docetaxel + ADT	ADT		OS
**STAMPEDE (ARM C)^28^**	November 2005-March 2013	Clarke et al.	1:2	III	Docetaxel + ADT	ADT		OS
**CHAARTED** ^3,40^	July 2006-November 2012	Sweeney et al.^2^ Kyriakopoulos et al.^27^	1:1	III	Docetaxel + ADT	ADT		OS
**LATITUDE^5^ ^,^ ^29^**	February 2013-December 2014	Fizazi et al.^4^ Fizazi et al.^28^	1:1	III	Abiraterone + PDN + ADT	Placebo + ADT		rPFS, OS
**STAMPEDE (ARM G)^6^**	2011-2014	James et al.	1:1	III	Abiraterone + PDN + ADT	ADT		OS
**TITAN^7^**	December 2015-July 2017	Chi et al.	1:1	III	Apalutamide + ADT	Placebo + ADT	Docetaxel for a maximum of 6 cycles before randomization	rPFS, OS
**ENZAMET^8^ ^,^ ^30^**	March 2014-March 2017	Davis et al.^8^ Davis et al.^31^	1:1	III	Enzalutamide + ADT	NSAA + ADT	Docetaxel for a maximum of 6 cycles, up to 2 cycles were permitted before randomization.	OS
**ARCHES^9^**	January 2014-January 2017	Armstrong et al.	1:1	III	Enzalutamide + ADT	Placebo + ADT	Docetaxel for a maximum of 6 cycles before randomization	rPFS
**ARANOTE^10^**	February 2021-June 2024	Saad et al.	2:1	III	Darolutamide + ADT	Placebo + ADT		rPFS
**PEACE-1^15^**	November 2013-December 2018	Fizazi et al.	1:1:1:1 in a 2x2 factorial design	III	Abiraterone + Docetaxel + ADT	Docetaxel + ADT	Radiation therapy to the primary tumor (for low metastatic burden)	rPFS, OS
**ARASENS^14^**	November 2016-June 2018	Smith et al.	1:1	III	Darolutamide + Docetaxel + ADT	Placebo + Docetaxel + ADT		OS

Abbreviations: ADT, androgen deprivation therapy; NSAA, non–steroidal anti–androgen drug; OS, overall survival; PDN, prednisone; rPFS, radiographic progression free survival.

**Table 2.** Characteristics of enrolled patients and efficacy data.

Trial (treatment strategy)		Patients enrolled, number	Age, years (range)	Age median, years (IQR)	Volume of metastatic disease, *n* (%)		Onset of the metastatic disease, *n* (%)		OS HR (95% CI)	rPFS HR (95% CI)	Median follow-up, months
					Low	High	Recurrent	De novo			
**GETUG-AFU 15**	Docetaxel + ADT (Doublet_CHEMO)	192	63 (57-68)		100 (52)	92 (48)	62 (33)	128 (67)	0.88 (0.68-1.14)	0.69 (0.55-0.87)	83.9
	ADT	193	64 (58-70)		102 (53)	91 (47)	46 (24)	144 (76)			
**STAMPEDE (ARM C)^a^**	Docetaxel + ADT (Doublet_CHEMO)	362		65 (62-70)	124 (46)	148 (54)	15 (3)	347 (59)	0.81 (0.69-0.95)		78.2
	ADT	724		65 (60-71)	238 (43)	320 (57)	34 (3)	690 (58)			
**CHAARTED**	Docetaxel + ADT (Doublet_CHEMO)	397	64 (36-88)		134 (33.8)	263 (66.2)			0.72 (0.59-0.89)		53.7
	ADT	393	63 (39-91)		143 (36.4)	250 (63.6)					
**LATITUDE**	Abiraterone + PDN + ADT (Doublet_ARPI)	597	68 (38-89)				0 (0)	697 (100)	0.66 (0.56-0.78)	0.47 (0.39-0.55)	41.4 for OS 30.4 for rPFS
	Placebo + ADT	602	67 (33-92)				0 (0)	602 (100)			
**STAMPEDE (ARM G)^a^**	Abiraterone + PDN + ADT (Doublet_ARPI)	500							0.61 (0.49-0.75)		40
	Placebo + ADT	502									
**TITAN**	Apalutamide + ADT (Doublet_ARPI)	525	69 (45-94)		200 (38.1)	325 (61.9)	85 (16.2)	411 (78.3)	0.67 (0.51-0.89)	0.48 (0.39-0.60)	44.0
	Placebo + ADT	527	68 (43-90)		192 (36.4)	335 (63.6)	59 (11.2)	441 (83.7)			
**TITAN-TXT**	Apalutamide + Docetaxel + ADT (triplet)	58							1.27 (0.52-3.09)	0.47 (0.22-1.01)	44.0
	Placebo + Docetaxel + ADT	55									
**ENZAMET**	Enzalutamide + ADT (Doublet_ARPI)	563		69.2 (63.2- 74.5)	272 (48.3)	291 (51.7)	238 (42.3)	325 (57.7)	0.70 (0.58-0.84)		68.0
	NSAA + ADT	562		69.0 (63.6-74.5)	265 (47.2)	297 (52.8)	235 (41.8)	327 (58.2)			
**ENZAMET-TXT**	Enzalutamide + Docetaxel + ADT (triplet)	254			177 (69.7)	77 (30.3)			0.82 (0.63-1.06)		
	NSAA + Docetaxel + ADT	249			179 (71.9)	70 (28.1)					
**ARCHES**	Enzalutamide + ADT (Doublet_ARPI)	574	70 (46-92)		220 (38.3)	354 (61.7)	83 (14.5)	402 (70.0)	0.81 (0.53-1.25)	0.39 (0.30-0.50)	44.6
	Placebo + ADT	576	70 (42-92)		203 (35.2)	373 (64.8)	86 (14.9)	365 (63.4)			
**ARCHES-TXT**	Enzalutamide + Docetaxel + ADT (triplet)	103							0.74 (0.46-1.20)	0.52 (0.30-0.89)	
	Placebo + Docetaxel + ADT	102									
**ARANOTE**	Darolutamide + ADT (Doublet_ARPI)	446	70 (43-93)		131 (29.4)	315 (70.6)	317 (71.1)	317 (71.1)	0.81 (0.59-1.12)	0.54 (0.41-0.71)	25.3
	Placebo + ADT	223	70 (45-91)		66 (29.6)	157 (70.4)	100 (22.4)	168 (75.3)			25.0
**PEACE-1**	Abiraterone*+ Docetaxel + ADT (triplet)	583		66 (60-70)	131 (37)	224 (63)	0 (0)	583 (100)	0.82 (0.69-0.98)	0.54 (0.41-0.71)	52.0
	Placebo + Docetaxel + ADT	589		66 (59-70)	123 (35)	232 (65)	0 (0)	589 (100)			
**ARASENS**	Abiraterone/PDN + Docetaxel + ADT (triplet)	651	67 (41-89)		154 (23.7)	497 (76.3)	86 (13.2)	558 (85.7)	0.68 (0.57-0.80)		43.7
	Placebo + Docetaxel + ADT	654	67 (42-86)		146 (22.3)	508 (77.7)	82 (12.5)	566 (86.5)			42.4

Abbreviations: CI, confidence interval; cPFS, clinical progression free survival; HR, hazard ratio; IQR, interquartile range; OS, overall survival, rPFS, radiographic progression free survival.

a Only the M1 patient’s subgroup of STAMPEDE trial was included in the present analysis.

OS was the primary endpoint in 6 of the 11 trials and rPFS was primary endpoint in 5 trials, as single primary endpoint in 2/5 and as a co-primary endpoint alongside OS in 3/5 (Table 1).

The overall quality of the studies included was confirmed to be high, further strengthening the reliability and robustness of the results obtained (Figure S3).

There was no evidence of significant inconsistency detected (*Q* = 7.05, df = 6, *P* = .3161). Local consistency was also robust, with no significant differences observed between direct and indirect estimates. The comparison between Doublet_CHEMO and Doublet_ARPI relied exclusively on indirect evidence (direct proportion = 0) (Table S3).

### OS

#### High-volume mHSPC

**Figure 1.**
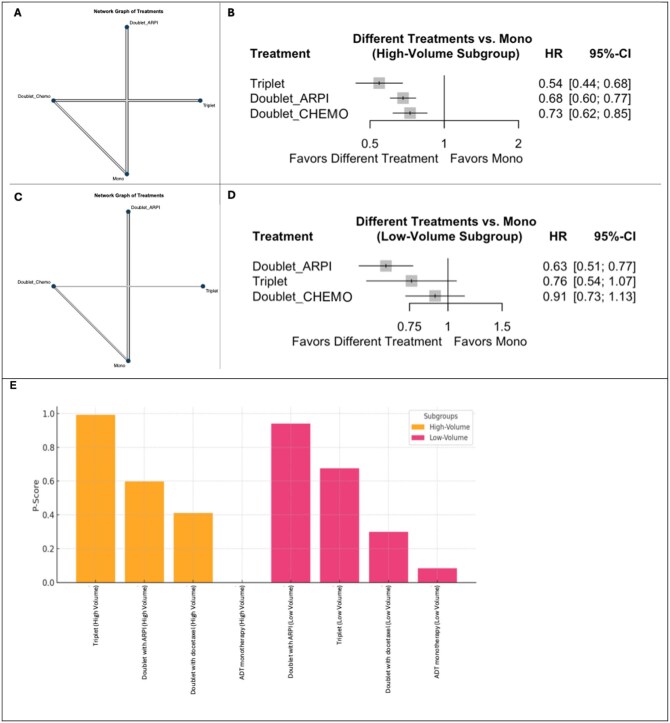
Network diagrams (A—high-volume patients; C—low-volume patients), forest plot of the fixed-effects model comparing treatments against ADT monotherapy (B—high-volume patients; D—low-volume patients), and treatment rankings based on *P*-scores (E) for OS. (A, C) The netgraphs illustrate the relationships between treatment strategies included in the network meta-analysis. Nodes represent the treatment strategies: Mono = ADT monotherapy; Doublet_CHEMO = ADT + docetaxel; Doublet_ARPI = ADT + ARPI; triplet = ADT + ARPI + docetaxel. Edges indicate direct comparisons between treatment strategies (the thickness of the edges is proportional to the number of studies comparing each pair of treatments). (B, D) Different treatment strategies are compared with the reference treatment in the forest plots. (E) *P*-scores for treatment rankings under the fixed effects model: Treatments are ranked based on their relative efficacy, with higher *P*-scores indicating a higher likelihood of being the most effective treatment. Abbreviations: ADT, androgen deprivation therapy; ARPI, androgen receptor pathway inhibitor; CI, confidence interval; HR, hazard ratio; OS, overall survival.

The network that reported OS data included 11 studies comparing four different treatment strategies with ADT monotherapy serving as reference treatment comparator (see Figure 1).

In indirect comparison to ADT monotherapy among patients with high-volume mHSPC, triplets showed the strongest OS-improvement with a 46% risk reduction of death (HR: 0.54; 95% CI: 0.44-0.68, *P* < .0001). Doublet_ARPI also improved OS significantly (HR: 0.68; 95% CI: 0.60-0.77; *P* < .0001), followed by Doublet_CHEMO showing a 27% reduction in the risk of death (HR: 0.73; 95% CI: 0.62-0.85; *P* < 0.0001) (Figure 1B).

Treatment ranking based on *P*-scores revealed triplets as the most effective treatment strategy, with a *P*-score of 0.99, Doublet_ARPI ranked second (*P*-score 0.59) and Doublet_CHEMO third (*P*-score 0.41) while ADT alone was least effective (*P*-score 0.00). Consequently, triplets achieve the best OS in patients with high-volume mHPSC (Figure 1E).

Interestingly, comparing triplets to Doublet_ARPI only showed a trend towards improved OS (HR: 0.80; 95% CI: 0.63-1.03), but this difference was not statistically significant. When compared to Doublet_CHEMO instead, triplets demonstrated a significant OS-benefit (HR: 0.75; 95% CI: 0.64-0.87) (Table S4 and Figure S4).

#### Low-volume mHSPC

Data on OS among low-volume mHSPC patients were available from 11 studies (Figure 1C). Again, ADT monotherapy served as reference treatment for collective treatment efficacy analysis.

Pairwise OS comparisons revealed significant differences between treatments in the low-volume subgroup. Interestingly, Doublet_ARPI was the only treatment modality to demonstrate superior efficacy over ADT alone with 37% reduction of the risk of death (HR: 0.63; 95% CI: 0.51-0.77). Both Doublet_CHEMO (HR: 0.91; 95% CI: 0.73-1.13) and triplet treatments (HR: 0.76; 95% CI: 0.54-1.07) numerically improved OS but without reaching statistical significance when compared to ADT alone.

The critical comparisons between triplet and doublet approaches yielded mixed results, with a HR of 1.20 for triplet when compared to Doublet_ARPI (95% CI: 0.82-1.79) and a HR of 0.84 (95% CI: 0.65-1.08) when compared to Doublet-CHEMO with no comparison reaching statistical significance (Table S5 and Figure S5).

As per treatment ranking, Doublet_ ARPI emerged as the most beneficial treatment for low-volume mHSPC patients (*P*-score, 0.94). Triplets ranked (*P*-score, 0.68), followed by Doublet_CHEMO (*P*-score, 0.29), which showed very limited activity in this patient subgroup. Monotherapy with ADT was again the least active treatment (0.08) with regards to OS (Figure 1E).

### OS subgroup analyses stratified by volume and disease onset

**Figure 2.**
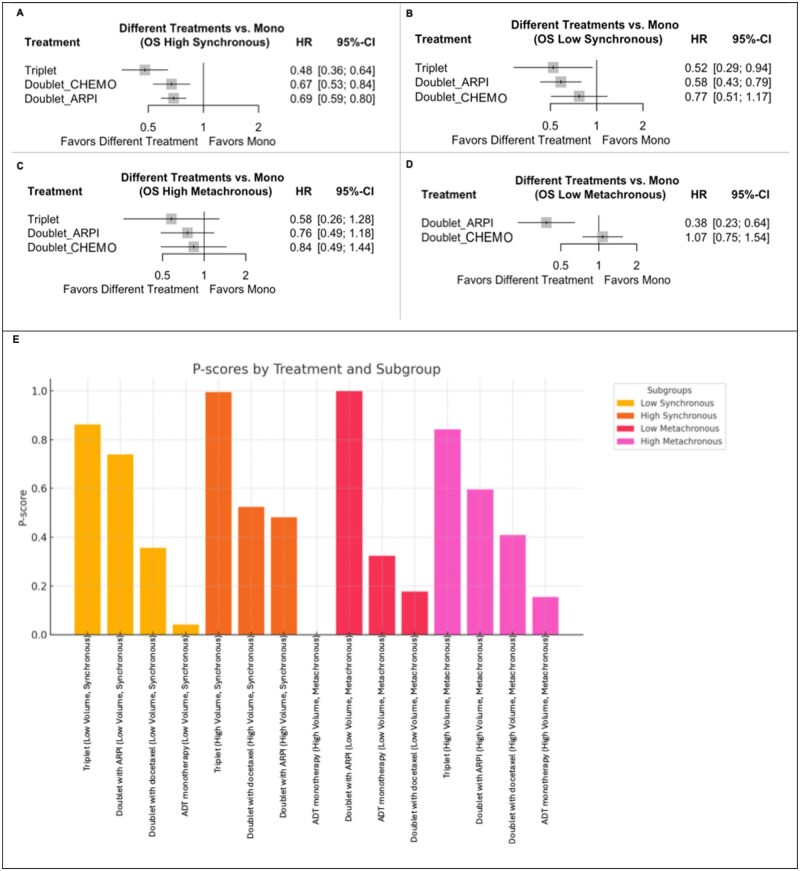
Forest plot of the fixed-effects model comparing treatments against ADT monotherapy in: high-volume synchronous patients (A), low-volume synchronous patients (B), high-volume metachronous patients (C), low-volume metachronous patients (D), and treatment rankings based on *P*-scores (E) for OS. (A-D) Different treatment strategies compared to reference ADT monotherapy as shown in the forest plots. (E) *P*-scores for treatment rankings under the fixed effects model: Treatments are ranked based on their relative efficacy, with higher *P*-scores indicating a higher likelihood of being the most effective treatment. Abbreviations: ADT, androgen deprivation therapy; ARPI, androgen receptor pathway inhibitor; CI, confidence interval; HR, hazard ratio; OS, overall survival.

Subsequently, patients were grouped according to disease volume and onset of metastasis for further OS subgroup analyses as follows: (1) High-volume/synchronous; (2) low-volume/synchronous; (3) high-volume/metachronous; (4) low-volume/metachronous (Figure 2A-E).

#### High-volume/synchronous mHSPC subgroup

In the high-volume/synchronous subgroup, the network displayed robust connections for ADT alone, ADT + ARPI, and ADT + docetaxel, with limited data for triplets within 6 studies (Figure S6A). The forest plot of pairwise comparisons showed statistically significant improvements for both Doublet_ARPI (HR: 0.69; 95% CI: 0.59-0.80; *P* < .0001) and Doublet_CHEMO (HR: 0.67; 95% CI: 0.53-0.84; *P* = .0007), and a most prominent risk reduction of death by 52% with triplet therapy (HR: 0.48; 95% CI: 0.36-0.64; *P* < 0.0001) when compared to ADT Monotherapy (Figure 2A).

When compared to either of the 2 doublet combinations, triplets outperformed both, Doublet_CHEMO (HR: 0.71; 95% CI: 0.60-0.85) and Doublet_ARPI (HR: 0.70; 95% CI: 0.50-0.97) with an about 30% risk reduction of death and (Table S6 and Figure S7A).

This is also highlighted by the ranking analysis results with triplet ranked highest (*P*-score, 0.99), followed by Doublet_CHEMO (0.52), Doublet_ARPI (0.48), and ADT alone (0.0001) (Figure 2E).

#### Low-volume/synchronous mHSPC subgroup

Six studies were included in the network for the low-volume/synchronous patient subgroup (Figure S6B). As shown in the forest plot analysis, both triplets (HR: 0.52; 95% CI: 0.29-0.94, *P* = .03) and Doublet_ARPI (HR: 0.58; 95% CI: 0.43-0.79; *P* = .0006) treatments demonstrated an OS improvement compared to ADT alone (Figure 2B).

The comparison between triplets and doublet therapies did not show any significantly different activity of one over another, (Table S7, Figure S7B).

In the ranking based on *P*-scores triplet ranked highest (*P*-score, 0.86), followed by Doublet_ARPI (0.74), Doublet_CHEMO (0.36), and ADT monotherapy (0.04) (Figure 2E).

#### High-volume/metachronous mHSPC subgroup

In this subgroup, the network was limited to monotherapy ADT, Doublet_ARPI, and Doublet_CHEMO with only sparse and poorly connected data available for triplet combinations (Figure S6C). The pairwise comparisons indicated that none of the above achieved statistically significant OS improvement over the reference comparator, ADT alone (all *P* > .05, Figure 2C).

The efficacy of triplets in direct comparison against both doublet options was inconclusive (Table S8 and Figure S7C).

*P*-score ranking reflected the lack of conclusive results, with the triplet (*P*-score = 0.84) ranking highest, followed by Doublet_ARPI (0.59), Doublet_CHEMO (0.41), and ADT monotherapy (0.15) (Figure 2E).

#### Low-volume/metachronous mHSPC subgroup

In this analysis, no data on triplet treatment but only for doublets with ARPI or docetaxel were available (Figure S6D). The forest plot showed a clear and statistically significant advantage for Doublet_ARPI over ADT alone (HR: 0.38; 95% CI: 0.23-0.64; *P* = .0003). The comparison between Doublet_CHEMO and ADT monotherapy, instead, did not show any difference in OS impact (HR: 1.07; 95% CI: 0.75-1.54; *P* = .71) (Figure 2D, Table S9, and Figure S7D).

The ranking analysis had Doublet_ARPI ranked as top treatment strategy (*P*-score, 0.99), followed by ADT alone (0.32) and Doublet_CHEMO (0.18) (Figure 2E).

### rPFS analysis

**Figure 3.**
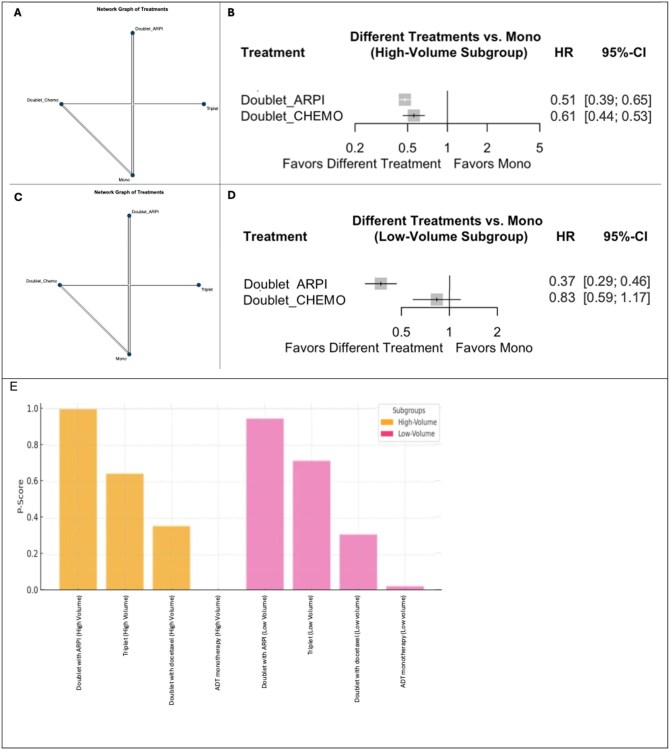
Network Diagrams (A—high-volume mHSPC; C—low-volume mHSPC), Forest plot of the fixed-effects model comparing treatments against ADT monotherapy (B—high-volume mHSPC; D—low-volume mHSPC), and treatment rankings based on *P*-scores (E) for rPFS. (A, C) The net graphs illustrate the relationships between treatment strategies included in the network meta-analysis. Nodes represent the treatment strategies: Mono = ADT monotherapy; Doublet_CHEMO = ADT + docetaxel; Doublet_ARPI = ADT + ARPI; triplet = ADT + ARPI + docetaxel. Edges indicate direct comparisons between treatment strategies (the thickness of the edges is proportional to the number of studies comparing each pair of treatments), with directed edges indicating the direction of the comparison. (B, D) Different treatment strategies are compared with the reference treatment in the forest plots. (E) *P*-scores for treatment rankings under the fixed effects model: Treatments are ranked based on their relative efficacy, with higher P-scores indicating a higher likelihood of being the most effective treatment for rPFS. Abbreviations: ADT, androgen deprivation therapy; ARPI, androgen receptor pathway inhibitor; 95% CI, 95% confidence interval; HR, hazard ratio; rPFS, radiographic progression-free survival.

In the secondary endpoint analysis of rPFS networks within high- and low-volume mHSPC patients, 4 treatment strategies were compared across five RCTs (Figure 3A and C).

The forest plot revealed that either doublet option, with ARPI and docetaxel, was associated with improved rPFS over ADT alone, with Doublet_ARPI achieving a more pronounced benefit with a 49% risk reduction for radiographic progression or death (HR: 0.51; 95% CI: 0.39-0.65) and a 39% risk reduction with Doublet_CHEMO treatment (HR: 0.61; 95% CI: 0.44-0.83) (Figure 3B, Table S10, and Figure S8).

Doublets were associated with better rPFS than ADT monotherapy also in the low-volume population, although the magnitude of effect varied between treatments. While Doublet_ARPI showed a significant benefit (HR: 0.37; 95% CI: 0.29-0.46), the impact of Doublet_CHEMO on rPFS was insignificant (HR: 0.83; 95% CI: 0.59-1.17) (Figure 3D, Table S11, and Figure S9).

The treatment rankings for rPFS were consistent across both fixed- and random-effect models. In both groups, Doublet_ARPI ranked highest (*P*-scores, 1.00 and 0.95), followed by triplets (0.64 and 0.72), Doublet_CHEMO (0.36 and 0.31), and ADT monotherapy (0.00 and 0.03) (Figure 3E).

## Discussion

Despite recent advances in the treatment of mHSPC, which were mainly derived from intensification of upfront combination treatment at onset of metastatic disease, questions remain regarding the optimal management in order to achieve best possible outcomes with the lowest possible treatment burden. Considering the lack of direct comparisons of the newest emerging combinations, for example, triplet regimens versus doublets with ADT plus ARPI, the aim of this project was to investigate the efficacy of all prospectively assessed systemic treatment options in the mHSPC setting, which were grouped as 4 different treatment strategies and stratified by uniformly available clinical characteristics, that is, disease volume (high vs. low) and onset of metastatic disease (synchronous vs. metachronous). Both factors are established predictors of survival for mHSPC patients, with high-volume/synchronous mHSPC patient showing the shortest OS due to aggressive disease and low-volume/metachronous mHSPC patients showing more indolent disease trajectories with longer survival. This network meta-analysis therefore provides the most up-to-date collated evidence on the performance of triplets, doublets, and ADT monotherapy, all of which are now available treatment strategies. Our results inform everyday practice by aiding treatment decision making for individual patients but also inform future clinical trial design.

Our analysis confirms the superior efficacy of triplet combinations to improve both OS and rPFS in mHSPC patients with high-volume disease in general, independent of the timing of metastatic disease onset. This benefit was clearly driven by treatment results achieved in patients with high-volume plus synchronous disease, whereas in the high-volume/metachronous subgroup, no clear survival advantage was observed for either triplet or doublet combination approaches over ADT alone. This uncertainty across studies likely reflects the considerable clinical and biological diversity within this population and highlights the need for future research to unravel patient heterogeneity, treatment response predictors and molecular subtypes to guide treatment decisions.

Unlike patients with *de novo* metastatic disease, patients with metachronous development of metastasis have typically received prior radical local treatment, surgery and/or radiotherapy, which may influence tumor evolution, clonal selection and subsequent responsiveness to systemic therapies.^32^ Importantly, the majority of metastatic cases arise as a progression from previously localized disease, whereas *de novo* or synchronous metastatic presentations represent a smaller subset. Yet, patients with metachronous metastases are often underrepresented in RCTs, reducing the statistical power to detect meaningful differences, and several ongoing prospective studies will provide further data regarding the role of triplet therapy in this large subgroup of patients. The phase III ASPIRE trial, (NCT06931340, Alliance Cooperative Group), for instance, is currently investigating whether adding docetaxel to ADT plus apalutamide improves outcomes in patients with metastatic hormone-sensitive carcinoma compared to ADT plus apalutamide alone, which serves as the control arm and stands out from prior triplet trials where the comparator was ADT plus docetaxel. The trial’s planned subgroup analyses based on disease volume, timing of metastases and molecular features may help understand whether chemotherapy intensification beyond ARSI-based doublets confers additional benefit, especially in biomarker- or volume-selected subgroups.^33^

On the contrary, in patient with low-volume mHSPC, ARPI-based doublets outperformed more intense triplets with added docetaxel. When stratified by disease onset, the comparison between triplets and doublets for the synchronous population did not yield statistically significant results. However, it should be noted that only the ENZAMET trial provided data on both disease volume and timing, and therefore, the assessable patient number was limited. More data are hence needed for more definite conclusions.

Similarly, for low-volume/metachronous mHSPC, the only trial results available for our analysis concerned doublet combinations, but no data on triplet treatment as these patients were excluded from PEACE-1 and ARASENS. According to our findings, ARSI-based doublets appear to offer the most favorable outcomes in this subgroup. Due to the aforementioned absence of triplets data, no conclusions can be drawn about the potential benefit of treatment intensification beyond doublet therapy in this population. Nevertheless, as recent data from a STAMPEDE ancillary study has shown, those within the low-volume mHSPC patients subgroup who had a high Decipher mRNA Score are likely to achieve poorer clinical outcomes.^34^ Therefore, molecular profiling may help identify particular subgroups that may benefit from intensified treatment regimens, also including docetaxel.

We acknowledge our study has some limitations. Since we adopted a networking approach, we had to account for heterogeneity risks across the trials, such as different inclusion criteria, follow-up duration, chosen efficacy endpoints, and different sample sizes across volume subgroups. We included the combination of darolutamide and ADT in the current analysis; however, it should be highlighted that median follow-up of the ARANOTE trial significantly differs from the other studies as it is the most recently presented RCT and thus some OS data OS are still immature and need to be interpreted cautiously. Nevertheless, we addressed such possible biases through consistency analyses and subgroup evaluation for the OS endpoint. In addition, we included the PEACE-1 trial, in which radiotherapy to the primary tumor was offered only to patients with a low metastatic burden, according to the trial’s 2 × 2 factorial design. In our analysis, however, the triplet arm comprises all patients treated with ADT, docetaxel and abiraterone—regardless of whether they received radiotherapy. Since the data did not allow us to separate patients based on radiotherapy exposure within this group, we acknowledge that radiotherapy could represent a potential unmeasured confounder, especially among patients with synchronous low-volume disease. Compared to other network meta-analyses we used a homogenous definition for PFS, including only rPFS as the secondary endpoint. Many RCTs included in this work presented radiographic, biochemical and/or clinical PFS data. By opting to analyze only rPFS, we achieved better data consistency, with low heterogeneity between the included RCTs, which is a differentiation from a recent network meta-analysis from Hoeh et al.^35^ In this work, the lack of grouping by doublets/triplets makes it difficult to derive conclusions on the best treatment strategy based on disease volume and onset, while authors also did not include triplets at all in their analysis stratified by disease burden.

Another limitation of our work is a lack of efficacy data for some subgroups, which limited the extension of study populations’ analyses, as well as the data included in the subgroup analyses we already conducted.

Furthermore, our network meta-analysis did not include other treatment strategies, such as PARP-inhibitor combinations or RLTs, as this was not our intention and scope of this analysis.^36–38^ Still, in the future, they may represent further options to be evaluated in this setting as they showed promising results in the castration-resistant setting.^39^^,^^40^ Notably, the disease burden in all the included RCTs has always been evaluated with conventional imaging. Therefore, no conclusions can be drawn from our analysis about the use of PET-PSMA and new imaging techniques for assessing the burden of disease and related treatment outcomes and expected survival.^41^^,^^42^ Moreover, our analysis focuses on efficacy data and does not assess toxicity or quality of life data, which also impact treatment decision-making for individual patients.

In conclusion, here we present the first systematic review of the literature and network meta-analysis on available treatment modalities in mHSPC specifically addressing the question of the best treatment options based on disease volume and onset of metastatic spread, which inevitably aids treatment decision making for mHSPC patients in a routine care setting.

## Supplementary Material

oyaf386_Supplementary_Data

## Data Availability

Data derive from already published manuscripts, and are available upon request to the corresponding author.
