# Supplementary material for: Best therapeutic approach in metastatic hormone-sensitive prostate cancer based on disease volume: a systematic review and network meta-analysis
Source: Oncologist. 2025 Nov 17;31(2):oyaf386. doi: 10.1093/oncolo/oyaf386 (PMC12832964; doi:10.1093/oncolo/oyaf386)
Supplement: oyaf386_Supplementary_Data [file oyaf386_supplementary_data.zip › Supplementary files_NeMA 19.01.docx]

**Supplementary Figure 1. PRISMA Checklist for systematic review and meta-analysis.**

| **Topic** | **No.** | **Item** | **Location where item is reported** |
| --- | --- | --- | --- |
| **TITLE** |  |  |  |
| **Title** | 1 | Identify the report as a systematic review. | Title page |
| **ABSTRACT** |  |  |  |
| **Abstract** | 2 | See the PRISMA 2020 for Abstracts checklist | Abstract |
| **INTRODUCTION** |  |  |  |
| **Rationale** | 3 | Describe the rationale for the review in the context of existing knowledge. | 1. Introduction |
| **Objectives** | 4 | Provide an explicit statement of the objective(s) or question(s) the review addresses. | 1. Introduction |
| **METHODS** |  |  |  |
| **Eligibility criteria** | 5 | Specify the inclusion and exclusion criteria for the review and how studies were grouped for the syntheses. | 2. Methods; 2.2 Inclusion and exclusion criteria |
| **Information sources** | 6 | Specify all databases, registers, websites, organizations, reference lists and other sources searched or consulted to identify studies. Specify the date when each source was last searched or consulted. | 2. Methods; 2.1 Data retrieval strategies |
| **Search strategy** | 7 | Present the full search strategies for all databases, registers and websites, including any filters and limits used. | 2. Methods; 2.1 Data retrieval strategies; Supplementary Table 1 |
| **Selection process** | 8 | Specify the methods used to decide whether a study met the inclusion criteria of the review, including how many reviewers screened each record and each report retrieved, whether they worked independently, and if applicable, details of automation tools used in the process. | 2. Methods; 2.3 Data extraction; Supplementary Table 2 |
| **Data collection process** | 9 | Specify the methods used to collect data from reports, including how many reviewers collected data from each report, whether they worked independently, any processes for obtaining or confirming data from study investigators, and if applicable, details of automation tools used in the process. | 2. Methods; 2.3 Data extraction and quality assessment |
| **Data items** | 10a | List and define all outcomes for which data were sought. Specify whether all results that were compatible with each outcome domain in each study were sought (e.g. for all measures, time points, analyses), and if not, the methods used to decide which results to collect. | 2. Methods; 2.3 Data extraction and quality assessment |
|  | 10b | List and define all other variables for which data were sought (e.g. participant and intervention characteristics, funding sources). Describe any assumptions made about any missing or unclear information. | 2. Methods; 2.3 Data extraction and quality assessment |
| **Study risk of bias assessment** | 11 | Specify the methods used to assess risk of bias in the included studies, including details of the tool(s) used, how many reviewers assessed each study and whether they worked independently, and if applicable, details of automation tools used in the process. | 2. Methods; 2.3 Data extraction and quality assessment |
| **Effect measures** | 12 | Specify for each outcome the effect measure(s) (e.g. risk ratio, mean difference) used in the synthesis or presentation of results. | 2. Methods; 2.4 Statistical analysis |
| **Synthesis methods** | 13a | Describe the processes used to decide which studies were eligible for each synthesis (e.g. tabulating the study intervention characteristics and comparing against the planned groups for each synthesis (item 5)). | 2. Methods; 2.3 Data extraction and quality assessment |
|  | 13b | Describe any methods required to prepare the data for presentation or synthesis, such as handling of missing summary statistics, or data conversions. | 2. Methods; 2.3 Data extraction and quality assessment |
|  | 13c | Describe any methods used to tabulate or visually display results of individual studies and syntheses. | 2. Methods; 2.3 Data extraction and quality assessment; 2.4 Statistical analysis |
|  | 13d | Describe any methods used to synthesize results and provide a rationale for the choice(s). If meta-analysis was performed, describe the model(s), method(s) to identify the presence and extent of statistical heterogeneity, and software package(s) used. | 2. Methods; 2.4 Statistical analysis |
|  | 13e | Describe any methods used to explore possible causes of heterogeneity among study results (e.g. subgroup analysis, meta-regression). | 2. Methods; 2.4 Statistical analysis |
|  | 13f | Describe any sensitivity analyses conducted to assess robustness of the synthesized results. | 2. Methods; 2.4 Statistical analysis |
| **Reporting bias assessment** | 14 | Describe any methods used to assess risk of bias due to missing results in a synthesis (arising from reporting biases). | 2. Methods; 2.3 Data extraction and quality assessment |
| **Certainty assessment** | 15 | Describe any methods used to assess certainty (or confidence) in the body of evidence for an outcome. | 2. Methods; 2.5 Assessment of evidence certainty |
| **RESULTS** |  |  |  |
| **Study selection** | 16a | Describe the results of the search and selection process, from the number of records identified in the search to the number of studies included in the review, ideally using a flow diagram. | 3. Results; 3.1 Search results and characteristics of the included studies; Supplementary Figure 2 |
|  | 16b | Cite studies that might appear to meet the inclusion criteria, but which were excluded, and explain why they were excluded. | 3. Results; 3.1 Search results and characteristics of the included studies; Supplementary Figure 2 |
| **Study characteristics** | 17 | Cite each included study and present its characteristics. | 3. Results; 3.1 Search results and characteristics of the included studies; Table 1; |
| **Risk of bias in studies** | 18 | Present assessments of risk of bias for each included study. | 3. Results; 3.1 Search results and characteristics of the included studies |
| **Results of individual studies** | 19 | For all outcomes, present, for each study: (a) summary statistics for each group (where appropriate) and (b) an effect estimate and its precision (e.g. confidence/credible interval), ideally using structured tables or plots. | 3. Results; 3.1 Search results and characteristics of the included studies; |
| **Results of syntheses** | 20a | For each synthesis, briefly summarise the characteristics and risk of bias among contributing studies. | 3. Results; 3.1 Search results and characteristics of the included studies; 3.2 rPFS; 3.3 OS; 3.4 OS subgroup analyses for volume and disease onset |
|  | 20b | Present results of all statistical syntheses conducted. If meta-analysis was done, present for each the summary estimate and its precision (e.g. confidence/credible interval) and measures of statistical heterogeneity. If comparing groups, describe the direction of the effect. | 3. Results; 3.2 rPFS; 3.3 OS; 3.4 OS subgroup analyses for volume and disease onset |
|  | 20c | Present results of all investigations of possible causes of heterogeneity among study results. | 3. Results; 3.2 rPFS; 3.3 OS; 3.4 OS subgroup analyses for volume and disease onset |
|  | 20d | Present results of all sensitivity analyses conducted to assess the robustness of the synthesized results. | 3. Results; 3.2 rPFS; 3.3 OS; 3.4 OS subgroup analyses for volume and disease onset |
| **Reporting biases** | 21 | Present assessments of risk of bias due to missing results (arising from reporting biases) for each synthesis assessed. | Supplementary Figure 3 |
| **Certainty of evidence** | 22 | Present assessments of certainty (or confidence) in the body of evidence for each outcome assessed. | 4.Discussion |
| **DISCUSSION** |  |  |  |
| **Discussion** | 23a | Provide a general interpretation of the results in the context of other evidence. | 4.Discussion |
|  | 23b | Discuss any limitations of the evidence included in the review. | 4. Discussion |
|  | 23c | Discuss any limitations of the review processes used. |  |
|  | 23d | Discuss implications of the results for practice, policy, and future research. | 4. Discussion |
| **OTHER INFORMATION** |  |  |  |
| **Registration and protocol** | 24a | Provide registration information for the review, including register name and registration number, or state that the review was not registered. | Title page |
|  | 24b | Indicate where the review protocol can be accessed, or state that a protocol was not prepared. | Title page |
|  | 24c | Describe and explain any amendments to information provided at registration or in the protocol. | Title page |
| **Support** | 25 | Describe sources of financial or non-financial support for the review, and the role of the funders or sponsors in the review. | Title page |
| **Competing interests** | 26 | Declare any competing interests of review authors. | Title page |
| **Availability of data, code and other materials** | 27 | Report which of the following are publicly available and where they can be found: template data collection forms; data extracted from included studies; data used for all analyses; analytic code; any other materials used in the review. | Title page |

**Supplementary Table 1. Search strategy for the databases used for the systematic review**

| **Database** | **Search string (**October 30, 2024) |
| --- | --- |
| PubMed | (“androgen receptor pathway inhibitor”[Title/Abstract] OR “androgen receptor signaling inhibitor”[Title/Abstract] OR androgen receptor targeted agent” [Title/Abstract] OR ARPI[Title/Abstract] OR ARSI[Title/Abstract] OR ARTA[Title/Abstract] OR abiraterone[Title/Abstract] OR apalutamide[Title/Abstract] OR darolutamide[Title/Abstract] OR enzalutamide[Title/Abstract] OR docetaxel"[Title/Abstract]) AND ("metastatic hormone sensitive prostate cancer"[Title/Abstract] OR "metastatic castration sensitive prostate cancer"[Title/Abstract] OR mCSPC[Title/Abstract] OR mHSPC[Title/Abstract])  Filters: English |
| Web of Science | ALL=(((androgen receptor pathway inhibitor) OR (androgen receptor signaling inhibitor) OR (androgen receptor targeted agent) OR (ARPI) OR (ARSI) OR (ARTA) OR (abiraterone) OR (apalutamide) OR (darolutamide) OR (enzalutamide) OR (docetaxel)) AND ((metastatic castration sensitive prostate cancer) OR (metastatic hormone sensitive prostate cancer) OR (mHSPC) OR (mCSPC))) |
| Scopus | (( “androgen receptor pathway inhibitor” OR “androgen receptor signaling inhibitor” OR “androgen receptor targeted agent” OR “ARPI” OR “ARSI” OR “ARTA” OR “abiraterone” OR “apalutamide” OR “darolutamide” OR “enzalutamide” OR “docetaxel”) AND ( “metastatic castration sensitive prostate cancer” OR “metastatic hormone sensitive prostate cancer” OR “mHSPC” OR “mCSPC”))  AND ( LIMIT-TO ( LANGUAGE , “English” ) ) |

**Supplementary Table 2. PICOS structure for study inclusion in the network meta-analysis**

| **Population** | Patients with mHSPC with high-volume and low-volume |
| --- | --- |
| **Interventions** | Triplet*, Doublet with ARPI**, Doublet with docetaxel, ADT monotherapy |
| **Comparator** | Doublet with ARPI**, Doublet with docetaxel, ADT monotherapy/PBO |
| **Outcome(s)** | OS (HR); rPFS (HR) |
| **Studies** | RCTs |

*Triplet included ADT + docetaxel + abiraterone/darolutamide, **Doublet with ARPI included ADT + abiraterone/apalutamide/darolutamide/enzalutamide

ADT: androgen deprivation therapy; ARPI: androgen receptor pathway inhibitor; HR: hazard ratio; mHSPC: metastatic hormone-sensitive prostate cancer; OS: overall survival; PBO: placebo; RCT: randomized clinical trial; rPFS: radiographic progression-free survival

# **Supplementary Table 3. Consistency Analysis Results**

## Global Consistency Test

| Statistic | Q | df | p-value | Tau | Tau² |
| --- | --- | --- | --- | --- | --- |
| Total | 7.05 | 6 | 0.3161 | - | - |
| Within Designs | 7.05 | 6 | 0.3161 | 0.0538 | 0.0029 |
| Between Designs | -0.00 | 0 | - | - | - |
| Doublet_CHEMO:Mono | 2.50 | 2 | 0.2860 | - | - |
| Doublet_ARPI:Mono | 4.55 | 4 | 0.3368 | - | - |

## Local Consistency Test (Node-Splitting)

| Comparison | k | Proportion Direct | HR (NMA) | HR Direct | HR Indirect |
| --- | --- | --- | --- | --- | --- |
| Doublet_CHEMO:Doublet_ARPI | 0 | 0 | 1.0678 | - | 1.0678 |
| Mono:Doublet_ARPI | 5 | 1.00 | 1.4699 | 1.4699 | - |
| Doublet_CHEMO:Mono | 3 | 1.00 | 0.7265 | 0.7265 | - |

**Supplementary Figure 2. PRISMA flow chart of the selection process**

**Supplementary Figure 3. Risk of Bias (ROB-2) analysis of the included studies**

|  | **Random sequence generation (selection bias)** | **Allocation concealment (selection bias)** | **Blinding of participants and personnel (performance bias)** | **Blinding of outcome assessment (detection bias)** | **Incomplete outcome data (attrition bias)** | **Selective reporting (reporting bias)** |
| --- | --- | --- | --- | --- | --- | --- |
| **ARANOTE** | **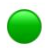** | **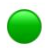** | **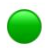** | **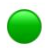** | 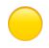 | **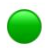** |
| **ARASENS** | **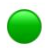** | **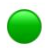** | **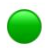** | **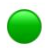** | **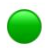** | **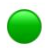** |
| **ARCHES** | **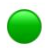** | **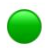** | **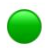** | **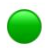** | **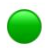** | **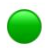** |
| **ENZAMET** | **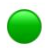** | **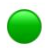** | 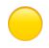 | **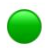** | **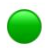** | **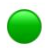** |
| **LATITUDE** | **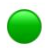** | **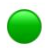** | **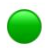** | **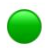** | **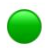** | **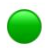** |
| **PEACE-1** | **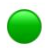** | **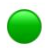** | 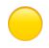 | **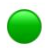** | **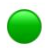** | **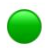** |
| **STAMPEDE**  **(Doce)** | **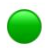** | **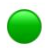** | 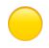 | **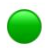** | **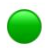** | **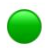** |
| **STAMPEDE (Abi)** | **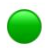** | **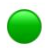** | **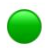** | **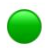** | **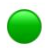** | **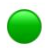** |
| **TITAN** | **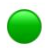** | **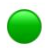** | **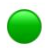** | **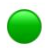** | **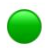** | **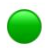** |
| **GETUG-AFU-15** | **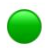** | **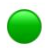** | 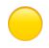 | **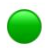** | 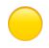 | **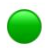** |
| **CHAARTED** | 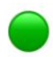 | **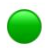** | 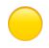 | 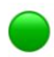 | **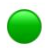** | **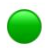** |

**Supplementary Table 4. League Table of HR and 95%CI for OS in the high-volume population**

|  | **Doublet_ARPI** | **Doublet_CHEMO** | **Triplet** | **Mono** |
| --- | --- | --- | --- | --- |
| **Doublet_ARPI** |  | 0.94  (0.77; 1.14) | 1.25  (0.97; 1.60) | 0.68  (0.60; 0.77) |
| **Doublet_CHEMO** | 1.06  (0.88; 1.30) |  | 1.33  (1.15; 1.55) | 0.73  (0.62; 0.85) |
| **Triplet** | 0.80  (0.63; 1.03) | 0.75  (0.64; 0.87) |  | 0.54  (0.44; 0.68) |
| **Mono** | 1.47  (1.30; 1.67) | 1.37  (1.18; 1.61) | 1.84  (1.48; 2.28) |  |

ARPI: androgen receptor pathway inhibitor; CI: confidence interval; HR: hazard ratio; rPFS: radiographic progression-free survival

**Supplementary Figure 4.** **Heatmap of HR and 95%CI for OS in the high-volume population**

ARPI: androgen receptor pathway inhibitor; CI: confidence interval; HR: hazard ratio; rPFS: radiographic progression-free survival

**Supplementary Table 5. League Table of HR and 95%CI for OS in the low-volume population**

|  | **Doublet_ARPI** | **Doublet_CHEMO** | **Triplet** | **Mono** |
| --- | --- | --- | --- | --- |
| **Doublet_ARPI** |  | 0.69  (0.51; 0.93) | 0.83  (0.56; 1.22) | 0.63  (0.51; 0.77) |
| **Doublet_CHEMO** | 1.45  (1.08; 1.96) |  | 1.19  (0.93; 1.54) | 0.91  (0.73; 1.13) |
| **Triplet** | 1.20  (0.82; 1.79) | 0.84  (0.65; 1.08) |  | 0.76  (0.54; 1.07) |
| **Mono** | 1.59  (1.30; 1.96) | 1.10  (0.89; 1.37) | 1.31  (0.94; 1.84) |  |

ARPI: androgen receptor pathway inhibitor; CI: confidence interval; HR: hazard ratio; OS: overall survival

**Supplementary Figure 5.** **Heatmap of HR and 95%CI for OS in the low-volume population**

ARPI: androgen receptor pathway inhibitor; CI: confidence interval; HR: hazard ratio; OS: overall survival

**Supplementary Figure 6. Network graphs of OS for: High-volume synchronous (A), Low-volume synchronous (B), high-volume metachronous (C), low-volume metachronous (D) patients**

| A. | 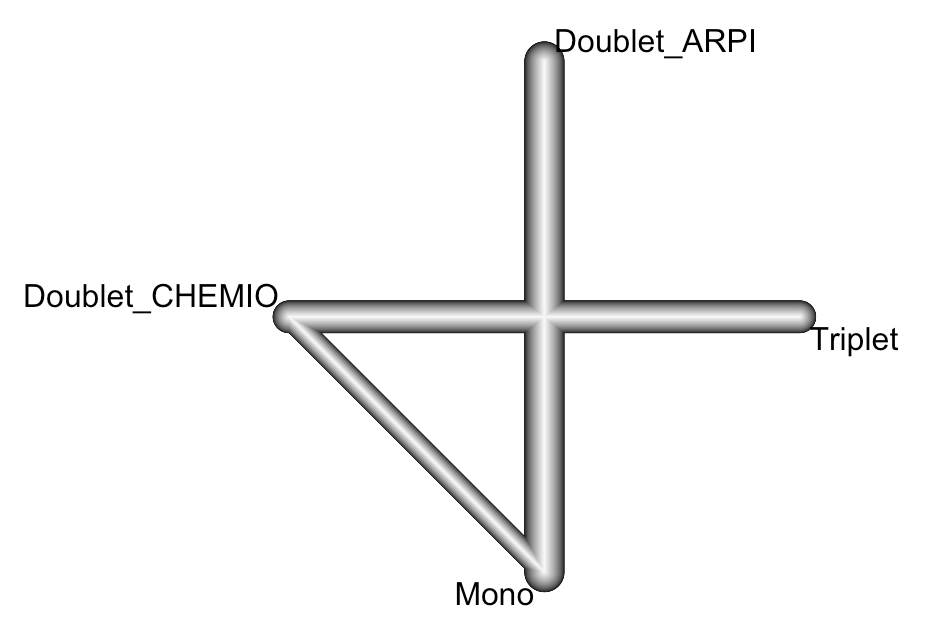 | B. | 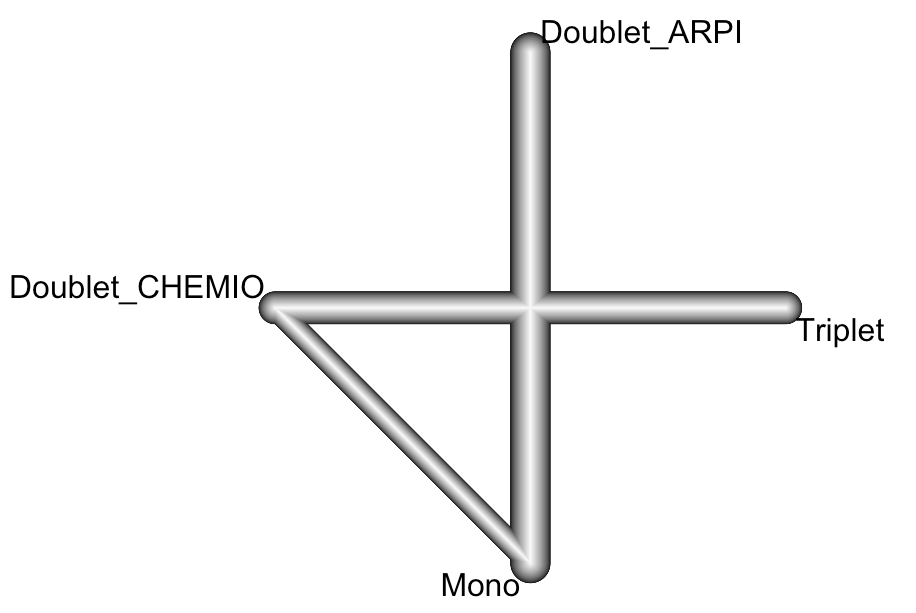 |
| --- | --- | --- | --- |
| C. | 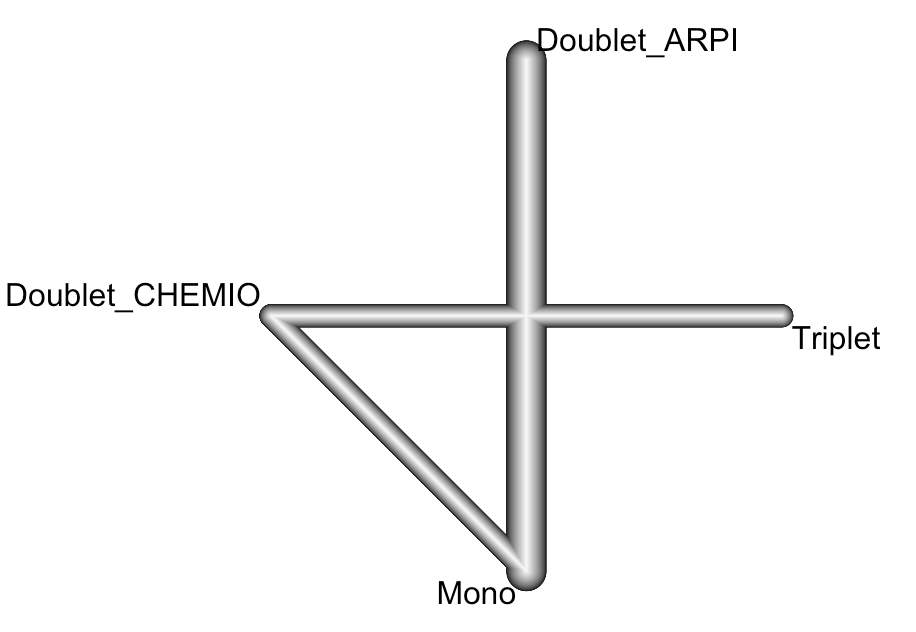 | D. | 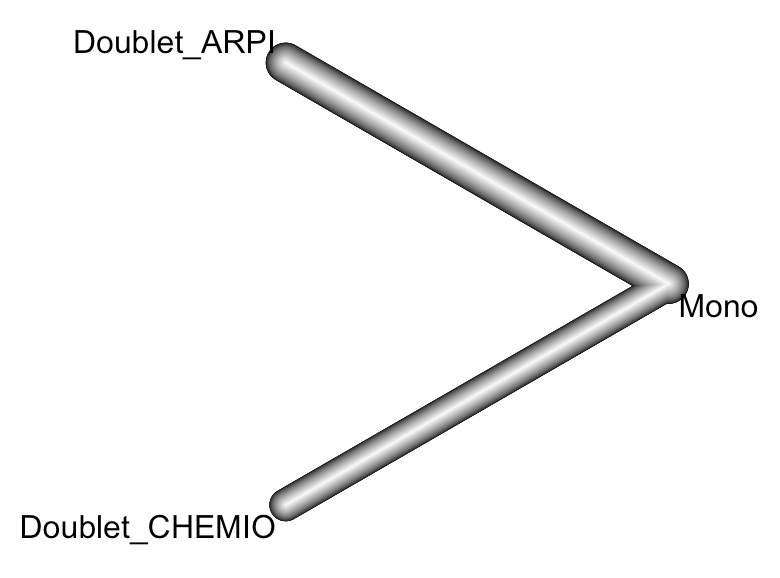 |

ARPI: androgen receptor pathway inhibitor

**Supplementary Figure 7. Heatmaps of OS for: High-volume synchronous (A), Low-volume synchronous (B), high-volume metachronous (C), low-volume metachronous (D) patients**

| **A** |  | **B** |  |
| --- | --- | --- | --- |
| **C** |  | **D** |  |

ARPI: androgen receptor pathway inhibitor; HR: hazard ratio; OS: overall survival

**Supplementary Table 6. League Table of HR and 95%CI for OS in the high-volume**

**synchronous population**

|  | **Doublet_ARPI** | **Doublet_CHEMO** | **Triplet** | **Mono** |
| --- | --- | --- | --- | --- |
| **Doublet_ARPI** |  | 1.03  (0.78; 1.35) | 1.43  (1.03; 1.99) | 0.69  (0.59; 0.80) |
| **Doublet_CHEMO** | 0.97  (0.74; 1.28) |  | 1.40  (1.18; 1.66) | 0.67  (0.53; 0.84) |
| **Triplet** | 0.70  (0.50; 0.97) | 0.71  (0.60; 0.85) |  | 0.48  (0.36; 0.64) |
| **Mono** | 1.45  (1.25; 1.70) | 1.50  (1.18; 1.89) | 2.09  (1.57; 2.78) |  |

ARPI: androgen receptor pathway inhibitor; HR: hazard ratio; OS: overall survival

**Supplementary Table 7. League Table of HR and 95%CI for OS in the low-volume**

**synchronous population**

|  | **Doublet_ARPI** | **Doublet_CHEMO** | **Triplet** | **Mono** |
| --- | --- | --- | --- | --- |
| **Doublet_ARPI** |  | 0.76  (0.45; 1.28) | 1.12  (0.58; 2.17) | 0.58  (0.43; 0.79) |
| **Doublet_CHEMO** | 1.32  (0.78; 2.22) |  | 1.48  (0.98; 2.23) | 0.77  (0.51; 1.17) |
| **Triplet** | 0.89  (0.46; 1.72) | 0.68  (0.45; 1.02) |  | 0.52  (0.29; 0.94) |
| **Mono** | 1.72  (1.27; 2.33) | 1.30  (0.99; 1.96) | 1.92  (1.07; 3.45) |  |

ARPI: androgen receptor pathway inhibitor; HR: hazard ratio; OS: overall survival

**Supplementary Table 8. League Table of HR and 95%CI for OS in the high-volume**

**metachronous population**

|  | **Doublet_ARPI** | **Doublet_CHEMO** | **Triplet** | **Mono** |
| --- | --- | --- | --- | --- |
| **Doublet_ARPI** |  | 0.90  (0.45; 1.82) | 1.31  (0.53; 3.24) | 0.76  (0.49; 1.18) |
| **Doublet_CHEMO** | 1.11  (0.55; 2.22) |  | 1.45  (0.81; 2.58) | 0.84  (0.49; 1.44) |
| **Triplet** | 0.76  (0.31; 1.89) | 0.69  (0.39; 1.23) |  | 0.58  (0.26; 1.28) |
| **Mono** | 1.32  (0.85; 2.04) | 1.19  (0.69; 2.04) | 1.73  (0.78; 3.81) |  |

ARPI: androgen receptor pathway inhibitor; HR: hazard ratio; OS: overall survival

**Supplementary Table 9. League Table of HR and 95%CI for OS in the low-volume**

**metachronous population**

|  | **Doublet_ARPI** | **Doublet_CHEMO** | **Mono** |
| --- | --- | --- | --- |
| **Doublet_ARPI** |  | 0.36  (0.19; 0.67) | 0.38  (0.23; 0.64) |
| **Doublet_CHEMO** | 2.78  (1.49; 5.26) |  | 1.07  (0.75; 1.54) |
| **Mono** | 2.63  (1.56; 4.35) | 0.93  (0.65; 1.33) |  |

ARPI: androgen receptor pathway inhibitor; HR: hazard ratio; OS: overall survival

**Supplementary Table 10. League Table of HR and 95%CI for rPFS in the high-volume population**

|  | **Doublet_ARPI** | **Doublet_CHEMO** | **Triplet** | **Mono** |
| --- | --- | --- | --- | --- |
| **Doublet_ARPI** |  | 0.86  (0.69; 1.05) | 2.08  (1.92; 2.33) | 0.48  (0.43; 0.52) |
| **Doublet_CHEMO** | 1.16  (0.95; 1.45) |  | 1.79  (1.50; 2.15) | 0.56  (0.46; 0.67) |
| **Triplet** | 0.48  (0.43; 0.52) | 0.56  (0.46; 0.67) |  | 0.28  (0.19; 0.42) |
| **Mono** | 2.08  (1.92; 2.33) | 1.79  (1.50; 2.15) | 3.52  (2.40; 5.16) |  |

ARPI: androgen receptor pathway inhibitor; CI: confidence interval; HR: hazard ratio; rPFS: radiographic progression-free survival

**Supplementary Figure 8.** **Heatmap of HR and 95%CI for rPFS in the high-volume population**

ARPI: androgen receptor pathway inhibitor; CI: confidence interval; HR: hazard ratio; rPFS: radiographic progression-free survival

**Supplementary Table 11. League Table of HR and 95%CI for rPFS in the low-volume population**

|  | **Doublet_ARPI** | **Doublet_CHEMO** | **Triplet** | **Mono** |
| --- | --- | --- | --- | --- |
| **Doublet_ARPI** |  | 0.44  (0.29; 0.67) | 0.75  (0.38; 1.49) | 0.37  (0.29; 0.46) |
| **Doublet_CHEMO** | 2.27  (1.49; 3.45) |  | 1.69  (0.98; 2.94) | 0.83  (0.59; 1.17) |
| **Triplet** | 1.33  (0.67; 2.63) | 0.59  (0.34; 1.02) |  | 0.49  (0.26; 0.94) |
| **Mono** | 2.70  (2.17; 3.45) | 1.20  (0.85; 1.69) | 2.03  (1.06; 3.89) |  |

ARPI: androgen receptor pathway inhibitor; CI: confidence interval; HR: hazard ratio; rPFS: radiographic progression-free survival

**Supplementary Figure 9.** **Heatmap of HR and 95%CI for rPFS in the low-volume population**

ARPI: androgen receptor pathway inhibitor; CI: confidence interval; HR: hazard ratio; rPFS: radiographic progression-free survival
